# Extra-tongue oral granular cell tumor: Histological and immunohistochemical aspect

**DOI:** 10.4317/medoral.21401

**Published:** 2016-12-06

**Authors:** Priscila-Lie Tobouti, Fernanda-Mombrini Pigatti, Maria-Carolina Martins-Mussi, Bruno-Tavares Sedassari, Suzana-Cantanhede Orsini-Machado de Sousa

**Affiliations:** 1 DDS, MSc, PhD. School of Dentistry, University of São Paulo, Oral Pathology department Av. Professor Lineu Prestes, 2227 Cidade Universitária 05508-000-São Paulo, SP, Brazil

## Abstract

**Background:**

Granular cell tumor (GCT) is an uncommon benign tumor founded in any part of the body but mainly in the tongue. Extra-tongue oral granular cell tumor (ETOGCT) is rare with few cases reported. Here we describe seven cases of oral GCT located in sites other then the tongue and discuss histopathological and immunohistochemical differences between differential diagnoses.

**Material and Methods:**

We retrieved all cases diagnosed with oral granular cell tumor, from the Oral Pathology Service at the School of Dentistry/ University of São Paulo, and excluded the ones sited in the tongue. Immunohistochemical staining anti-S100 was also performed.

**Results:**

The presented cases of Extra-tongue Oral Granular Cell Tumor (ETOGT) are composed by granular cells with intimately association with the adjacent tissue. Atypia and mitoses were not seen, and in most cases, the typical pseudoepitheliomatous hyperplasia was not observed.

**Conclusions:**

The importance of an adequate attention is to avoid misdiagnoses, since ETOGT is rare and the tricking histopathological findings could induce to it. All the cases can be differentiated from the tumors that has a granular cell proliferation through a morphological analysis and when needed, immunohistochemistry stain.

**Key words:**Abrikossoff`s tumor, granular cell tumor, oral cavity.

## Introduction

Granular cell tumor is an uncommon benign neoplasm (1) that can be found in any part of the body but usually found in skin, respiratory tract, breast, gastrointestinal tract and over 50% in head and neck, mainly the tongue (2). Extra-tongue oral granular cell tumors are rare (3,4).

According to the World Health Organization (WHO), granular cell tumor is a benign tumor of soft tissue and is thought to be of Schwann cell origin (5). It is composed of a poorly demarcated accumulation of plump granular cells, which are often intimately associated with skeletal muscle (6).

Oral granular cell tumor can develop at any age, but it is commonly found between the second and sixth decades of life (1,2).

Histologically, establishment of OGCT diagnosis is easy but it can be misdiagnosed if not well observed, especially, because this lesion often shows a pseudoepitheliomatous hyperplasia, and islands of epithelium can be seen in the connective tissue (2,7). This feature frequently leads to a wrong interpretation as squamous cell carcinoma (SCC) mainly when the tumor is located in an area where OSCC is more likely to occur. If carefully analyzed, besides the pseudoepitheliomatous hyperplasia, this tumor is composed by polygonal cells with eosinophilic granular cytoplasm and small nuclei (4). Invasion is commonly seen, however there is no atypia (8).

The treatment is complete resection of the tumor and no further complement is needed. Presently we describe seven cases of oral GCT located in sites other then the tongue and discuss histopathological and immunohistochemical differences between differential diagnoses.

## Material and Methods

- Ethics Statement

This study was approved by the Human Research Ethics Committee of the Dental School- University of São Paulo (protocol n.754-618).

- Retrieval of tumor samples

All cases of GCT diagnosed at the Oral Pathology Service at the School of Dentistry/ University of São Paulo in the interval between 2002-2015, were retrieved. Initially, 37 formalin-fixed paraffin embedded tumor samples were retrieved. Due to the aim of this study, all the biopsies sited in the tongue were excluded and thus, 7 samples were included. Information regarding site of the lesion, age, sex and clinical diagnosis were collected from patients` records. The study was approved by a local Ethical Committee.

- Immunohistochemistry

As a matter of illustration and to have a better view of the invasion of the tumor with adjacent tissues, immunohistochemical staining anti S100 was performed using LSAB Dako kit (Dako, Carpinteria, USA) on a 3mm section from OGCT whole tissue. In brief, sections were deparaffinized and for antigen retrieval, microwaved in sodium citrate buffer, pH 6.0 for 5 minutes (800 W, medium power) was used. The sections were then incubated for 30 minutes in 6% hydrogen peroxide/methanol (9) solution to quench endogenous peroxidase activity. Primary antibodies were diluted in Tris-bovine serum albumin (BSA) buffer and the sections were incubated with the antibodies for 30 minutes. Then, a secondary biotinilated antibody and streptavidin-biotin-peroxidase complex were incubated using the LSAB Dako kit (Dako, Carpinteria, USA). Diaminobenzidine was used as the chromogen, followed by counterstaining with Mayer’s hematoxylin.

All the slides were reviewed and reevaluated by three pathologists.

## Results

- Patients record

From 37 cases of OGCT, 30 were located in the tongue and only 7 cases (23.33%) were sited out of the tongue. Among the 7 cases, 2 were in the lips and 2 in retrocomissural area, one case was found in buccal mucosa, one in palate and one in vestibule.

Age range was between 34-60, mean age of 51 years old. Both sexes were almost equally affected, 4 women and 3 men. Caucasians were more affected, and the most frequent clinical diagnosis were fibroma and pleomorphic adenoma, given the site of lesion.

- Histology features

**Figure 1:**
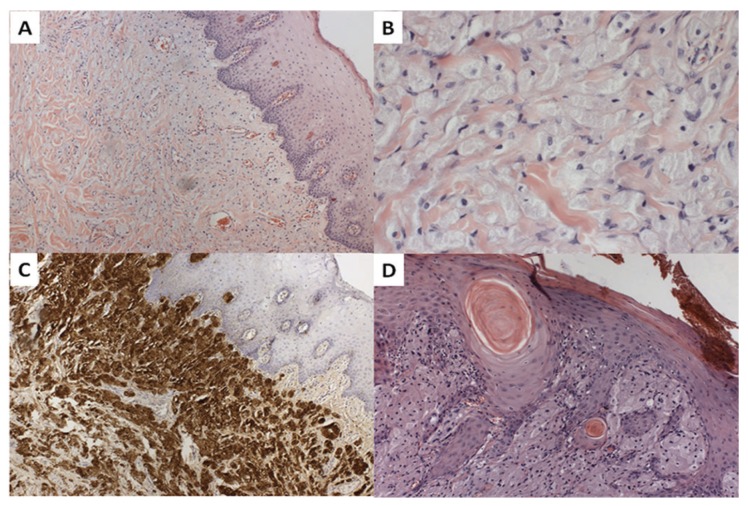
Histopathological characteristics of oral granular cell tumor: (A) Non-encapsulated tumor 100x. (B) Polygonal eosinophilic cells with central or peripheral small dark nuclei and abundant granular cytoplasm 400x. (C) Immunoreactive for S100 100x. (D) pseudoepitheliomatous hyperplasia 100x.

**Figure 2:**
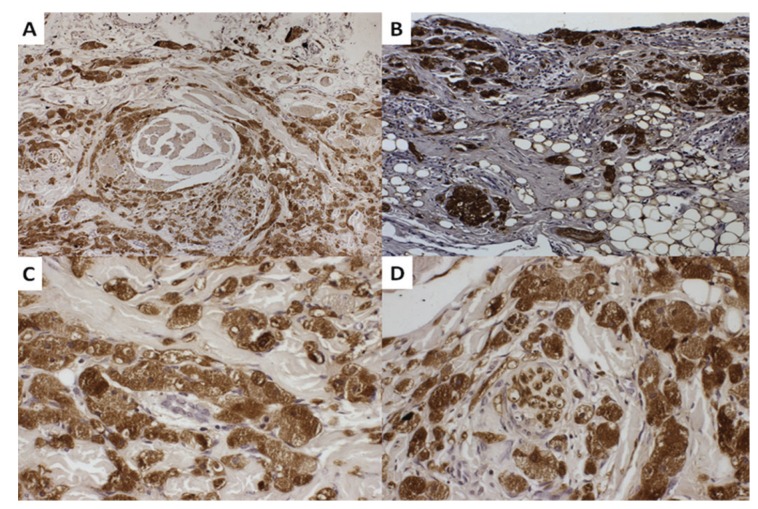
Invasion of the granular cells into: (A) Squeletal muscle 400x. (B) Adipocytes 100x. (C) Blood vessel 400x. (D) Nerve 400x.

Histologically, classic non-encapsulated tumor (Fig. 1A), composed mainly by polygonal eosinophilic cells with central small dark nuclei and abundant granular cytoplasm is seen (Fig. 1B). Those cells permeated the collagen fibers, in an intimate contact. Most cells were strongly positive to S100 (Fig. 1C). Invariably, all tumors showed invasion to the adjacent tissue, such as skeletal muscle, adipocytes, blood vessels and nerves (Fig. 2).

Only two of the cases showed pseudoepitheliomatous hyperplasia and one of these cases were characterized by proliferation of the epithelium with irregular nests and chords of squamous cells simulating an infiltrative proliferation into the connective tissue. Also, in the center of the nests, a formation of keratin pearl could be noticed, mimicking a squamous cell carcinoma feature. In the stroma a very mild presence of inflammation is seen. No atypical mitotic figures were observed (Fig. 1D).

## Discussion

Extra-tongue oral granular cell tumor (ETOGCT) is rare and can be misdiagnosed due to its histology. GCT was first described by Abrikossoff, in 1926 (10) and in 1931 (11), and named by him as “myoblastic myoma”. He described three cases in the tongue, one in the lip and one in the stomach and later, another 3 cases in the tongue and one in the skin were reported at a Congress (11).

At that time, Abrikossoff thought that this tumor was originated from muscle (11). However, studies of its histogenetic origin have shown that all OGCT are S-100 positive (12) and this protein is now used as protein-target for the diagnosis of this tumor. Other proteins such as p75, neuron-specific enolase (NSE) and CD68 are also immunoreactive favoring the nerve sheath differentiation (4).

The tongue is the most affected site (13,14), but this neoplasm can affect any part of the body and more than 50% of the cases are seen in the head and neck (13). Regarding the mean age and the equal incidence in both sexes, the present study is in agreement with the description from the WHO (5).

The present cases of ETOGCT did not show, in most cases, the typical pseudoepitheliomatous hyperplasia, which is the most striking feature that resembles SCC. The tumor is not encapsulated and the granular eosinophilic cells invade the adjacent tissue, but it does not characterize a malignant feature. Malignant transformation has been reported (15,16), and only 2% are capable to metastasize to distant sites (16). Histologically, the malignant counterpart shows a higher mitotic activity, necrosis, pleomorphism and a tendency to spindle cells component (16), features not seen in our cases.

Also, one important aspect that differentiates a GCT from a malignant tumor is the tumor stroma. It is known that the inflammatory tumor microenviroment has a key role in the cancer-promoting and cancer-antagonizing effects of inflammation (17,18). Thus the chronic inflammatory cells are intensively present in squamous cell carcinomas, but not seen in OGCT.

Some tumors mimic GCT thus showing a granular cell proliferation pattern while others that have granular cell components intermixed with the classic morphology of the tumor cell. The ones that have similar growth pattern as GCT are more difficult to diagnose, but some characteristics are different and immunohistochemistry can be helpful (4,19). Congenital epulis of newborn, leiomyoma, rhabdomyoma and oncocytoma can show a major presence of granular cell proliferation ( Table 1). Most of the papers highlight the fact that those tumors do not show pseudoepitheliomatous hyperplasia (20-22), as seen in GCT, but this statement is based on GCT located in the tongue. As we have shown here, most of our cases of extra-tongue GCT also did not show this feature.

**Table 1:**
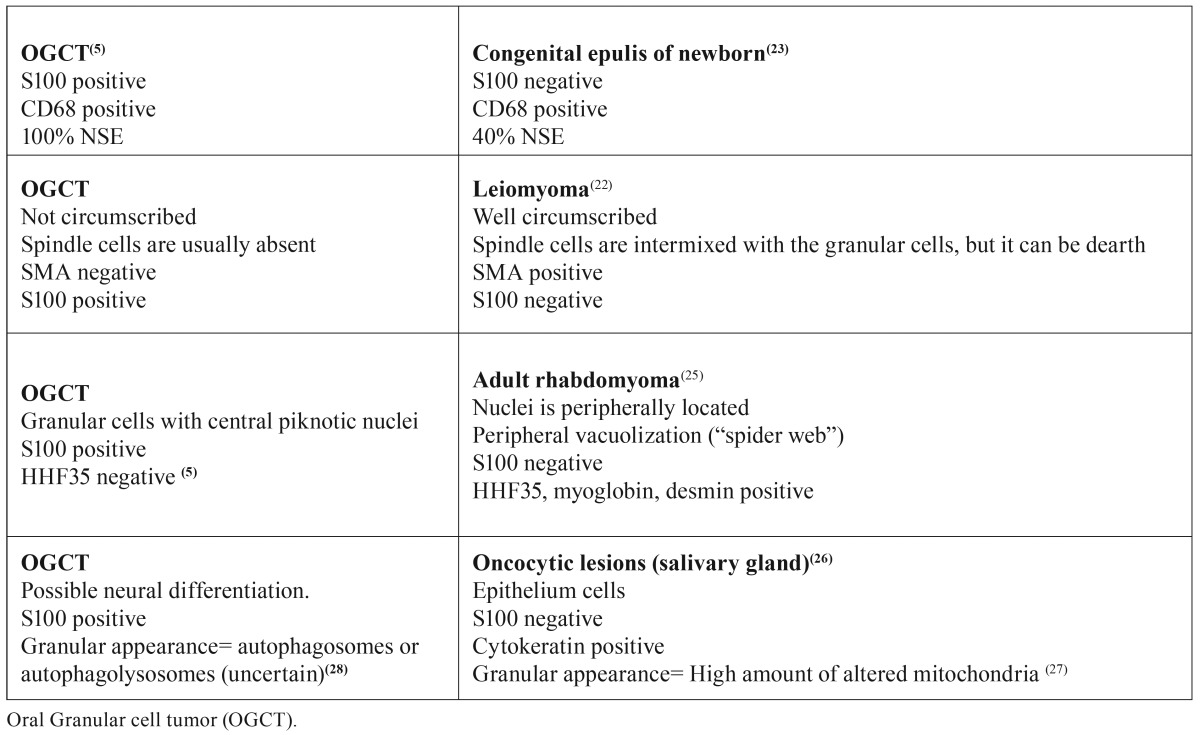
Comparison between oral granular cell tumor and tumors with predominantly granular cell components.

Congenital epulis of newborn (CEN) has been considered a reactive lesion rather than a true neoplasm. Both CEN and GCT express CD68 and almost 40% of CEN cases are immunoreactive for NSE (5,23). However, different from GCT it is not S100 positive (23,24).

Leiomyoma have a granular cell-subtype entity. This lesion is a smooth muscle proliferation so, invariably, it is smooth muscle actin positive, different from GCT. Also, morphologically it is a well-limited tumor and can show the presence of spindle cells intermixed with the granular cells (22). Features not seen in GCT. Another mesenchymal tumor with granular cell feature is the adult rhabdomyoma, which is a skeletal muscle neoplasm. In horizontal cross-section, muscle fibers show a polygonal shape with the appearance of abundant granular cytoplasm, but different from GCT cells, which show central nuclei, skeletal muscle show nuclei peripherally located. Also, a peripheral vacuolization, also called “spider web” is seen. Since it is a skeletal muscle tumor, it is immunopositive for HHF35, myoglobin and desmin (25).

Oncocytic cells seen in endocrine and exocrine tissues such as salivary gland are epithelial cells characterized by an abundant eosinophilic and granular cytoplasm with a central pyknotic nucleus (26). The “swollen” appearance is due to the high amount of mitochondrias (27), which are altered in the salivary gland tumors. Since the oncocytic cell is an epithelial cell, it will not stain for S100, as the granular cells from GCT are, thus, facilitating the diagnosis.

Schwanomas (28), melanocytic nevi (29), ameloblastomas (30), and metastatic lesions can also show a granular cell proliferation, but it is usually only a focus or an area of the tumor showing this feature ( Table 2).

**Table 2:**
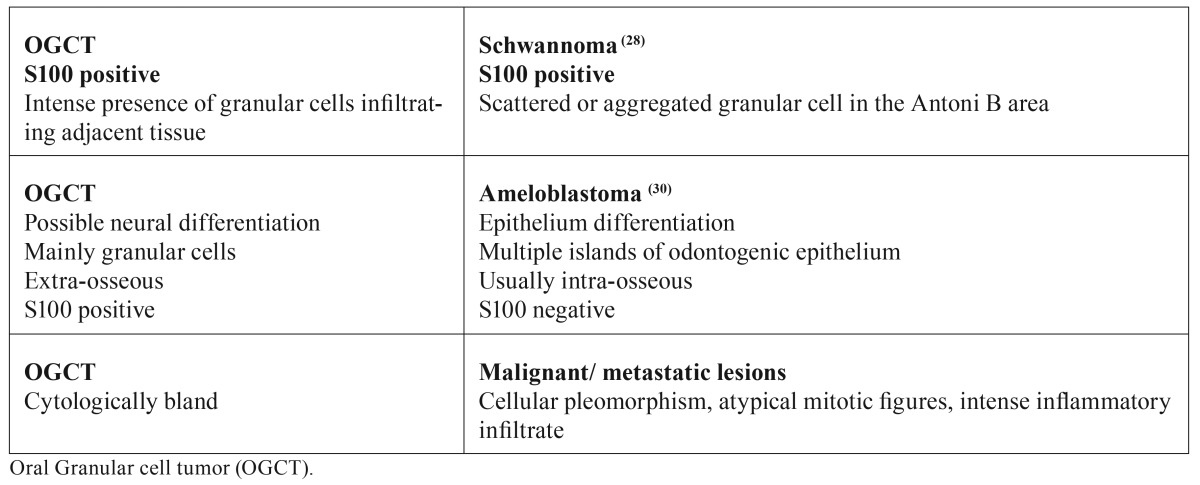
Comparison between oral granular cell tumor and tumors with a scattered granular cell component, mixed with the tumor cell proliferation.

We demonstrated a spectrum of pathologic findings seen in extra-tongue oral granular cell tumor and not seen in other benign and malignant lesions. The importance of an adequate attention is to avoid misdiagnoses, since ETOGT is rare and the tricking histopathological findings could induce to it. Microscopically, ETOGCT does not show major differences from the ones seen in the tongue and the most important aspect is the presence of an eosinophilic granular cell S100 positive, which characterizes the tumor.
